# Mapping the Specific Pathways to Early-Onset Mental Health Disorders: The “Watch Me Grow for REAL” Study Protocol

**DOI:** 10.3389/fpsyt.2020.00553

**Published:** 2020-06-19

**Authors:** Frances L. Doyle, Antonio Mendoza Diaz, Valsamma Eapen, Paul J. Frick, Eva R. Kimonis, David J. Hawes, Caroline Moul, Jenny L. Richmond, Divya Mehta, Sinia Sareen, Bronte G. Morgan, Mark R. Dadds

**Affiliations:** ^1^Faculty of Science, School of Psychology, University of Sydney, Sydney, NSW, Australia; ^2^Faculty of Medicine, School of Psychiatry, University of New South Wales, Sydney, NSW, Australia; ^3^Institute for Learning Sciences & Teacher Education, Australian Catholic University, Brisbane, QLD, Australia; ^4^Department of Psychology, Louisiana State University, Baton Rouge, LA, United States; ^5^Faculty of Science, School of Psychology, University of New South Wales, Sydney, NSW, Australia; ^6^School of Psychology and Counselling, Faculty of Health, Queensland University of Technology, Brisbane, QLD, Australia

**Keywords:** mental health, attention, responsiveness, learning, child, eye-tracking, infant, protocol

## Abstract

**Background:**

From birth, the human propensity to selectively attend and respond to critical super-stimuli forms the basis of future socio-emotional development and health. In particular, the first super-stimuli to preferentially engage and elicit responses in the healthy newborn are the physical touch, voice and face/eyes of caregivers. From this grows selective attention and responsiveness to emotional expression, scaffolding the development of empathy, social cognition, and other higher human capacities. In this paper, the protocol for a longitudinal, prospective birth-cohort study is presented. The major aim of this study is to map the emergence of individual differences and disturbances in the system of social-Responsiveness, Emotional Attention, and Learning (REAL) through the first 3 years of life to predict the specific emergence of the major childhood mental health problems, as well as social adjustment and impairment more generally. A further aim of this study is to examine how the REAL variables interact with the quality of environment/caregiver interactions.

**Methods/Design:**

A prospective, longitudinal birth-cohort study will be conducted. Data will be collected from four assessments and mothers' electronic medical records.

**Discussion:**

This study will be the first to test a clear developmental map of both the unique and specific causes of childhood psychopathology and will identify more precise early intervention targets for children with complex comorbid conditions.

## Background

Over 90% of Australian mental health expenditure is on adults (1), but most mental health problems begin very early in life (2). Close to 100% of referrals to mental health services for children are accounted for by four groups of disorders (in rough order of prevalence): disruptive behavior disorders (DBD), anxiety disorders, attention deficit hyperactivity disorder (ADHD), and autism spectrum disorders [ASD; (2)]. Early intervention programs are generally cost-effective in significantly reducing lifetime impairments of these disorders (3–5). This is a major achievement of the health sciences, but is offset by evidence that the most effective interventions only produce clinically significant change in around 50% of cases, such as parent training for DBD, (6, 7) and cognitive-behavior therapy for anxiety disorders (8–10). Moreover, outcomes are considerably worse for children with multiple comorbid problems, particularly those with symptoms of ADHD and ASD (11–14).

Early onset of child mental health problems (before age 10) is associated with chronically poor social adjustment, as well as psychological and physical health disorders (2). For example, research has shown that childhood DBD and anxiety/depressive disorders are the most reliable precursor of all types of adult mental health issues (2, 15). Despite these known associations, research into the major diagnoses of childhood is still largely a “causal-free zone” that says little about the specific pathways disorders take early in life, and the treatment needs of individual children. Further, the idea that these major childhood disorders are discrete categories has long been abandoned, as rates and patterns of co-occurrence or “comorbidity” are far higher than could exist by chance (16). Existing nosologies identify conditions that are not distinct disorders but are instead varied presentations of underlying syndromes (17, 18).

The search for environmental conditions that differentiate the common childhood disorders has yielded little fruit. That is, the major environmental risk factors identified for criminality, mental health disorders, substance use, and even physical health problems (e.g., child abuse, family disruption), appear to be largely non-specific rather than unique to particular disorders. In contrast, variations in prognosis and treatment response can be predicted by variations in critical neurodevelopmental systems associated with socio-emotional attention and responsiveness (19, 20). For example, DBD are commonly, but not always, associated with high emotional lability that specifies risk of developing anxiety, depression, and substance-use problems (2). Children with high emotional lability typically show “hot-tempered” or reactive aggression that develops largely through poor parenting and emotional dysregulation (21); these children also respond well to evidence-based treatments (6). Comparatively, children with low emotionality (or callous-unemotional (CU) traits) have relatively higher genetic influence, and respond relatively poorly to treatment (22). The example of DBD highlights the potential importance of tracking causal processes that may underlie multiple disorders (e.g., high emotional lability), as well identifying different causal processes (e.g., high emotional lability versus low emotionality) to the same outcome (e.g., early onset DBD). Due to of these differences, we separate DBD with elevated and non-elevated CU traits throughout this paper (hereafter designated as DBD+CU and DBD-CU, respectively).

### The REAL Model

**Figure 1:**
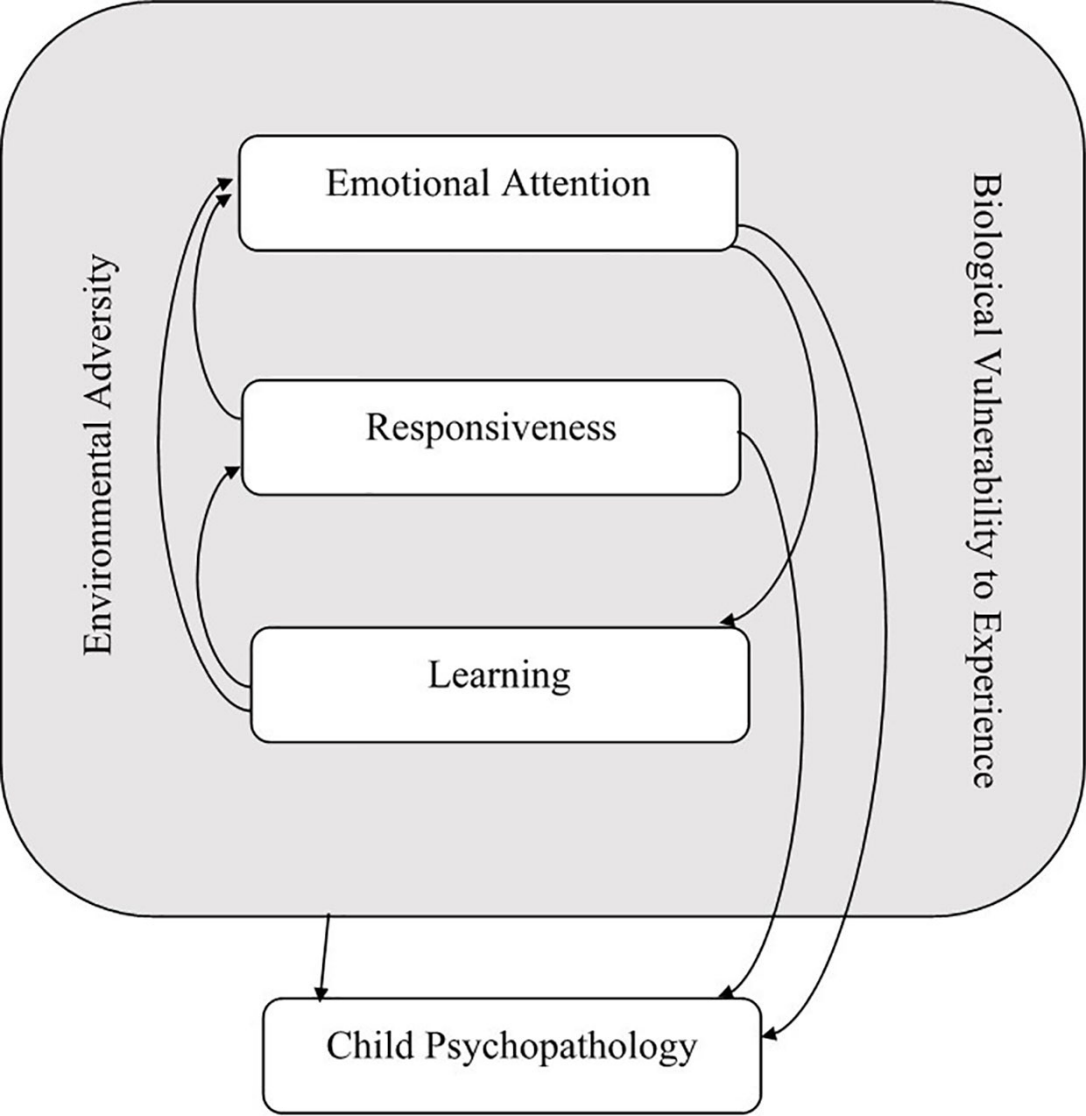
The REAL Model.

In the REAL model (see **Figure 1**), it is proposed that a critical organizing construct for identifying varying “trans-diagnostic” causal pathways and treatment needs in early-onset mental health problems involves individual differences in emotional attention and its corollaries of socio-emotional responsiveness and learning [which will be referred to as the REAL variables, i.e. Responsiveness, Emotional Attention, and Learning; (23)]. Socio-emotional attention refers to selectively attending to socio-emotional cues produced by other people (24, 25). In the case of young children, this is best operationalized as selective attention to the face/eyes of others that can be indexed using eye gaze tracking (26–28). Socio-emotional responsiveness refers to a child's visceral-behavioral responding to the emotional cues of other people (29, 30). Again, in infancy this first emerges as reciprocated facial emotional expression, gaze following, or joint attention, as well as intentional communication (26, 27, 29–31). Socio-emotional learning is defined as a child's propensity to show conditioned responses to (previously neutral) stimuli when paired with socio-emotional cues (32).

In the REAL model, it is proposed that individual deficits and excesses in each of these specific vulnerabilities underlie, and in part explain, hitherto inexplicable variations in early-onset mental health disorders (23). It is proposed in this model that problems with socio-emotional attention, as a deficit or excess, amplifies risk for psychopathology *via* the formation of (“vicious”) circular relationships between socio-emotional attention and responsiveness (or emotionality) and learning (25, 33–35). Although socio-emotional attention deficits or excesses can independently amplify psychopathology risk, it is also evident that the REAL constructs are interdependent. For example, socio-emotional responsiveness drives preferential attention to threat stimuli, which in turn, escalates responsiveness (36, 37). Socio-emotional responsiveness (and attention) also facilitate conditioned learning, whereby unconditioned “super”-stimuli drive (or fail to drive) aversive conditioning to common neutral stimuli (32, 38–40).

Moreover, each REAL variable needs to be considered against the child's capacity or propensity for attention, responsiveness, and learning to non-emotional stimuli (23). In the case of ASD, it is proposed that the REAL impairments also occur with regard to coordinated interpersonal processes that involve attending and responding to other people's gaze (31). Thus, it is essential to also examine constructs of attention to “self” stimuli, and joint attention alongside the REAL variables (23).

### Existing Evidence of the REAL Variables Predicting Psychopathology

#### Socio-Emotional Attention

Clear evidence shows that various forms of psychopathology are associated with individual differences in selective attention to critical emotional stimuli, including computerized emotional faces, sounds, and postures (41–46). These differences are most important when expressed early in development as a failure to attend to the emotions of attachment figures (47). Disrupted selective attention can drive cascading errors in the development of social cognition, empathy, and conscience (25, 34, 35, 42, 48). The emotional face is a super-stimulus (24, 49) that automatically sets off specific neurodevelopmental systems central to human development. Stimuli involving faces and eyes are therefore widely used to investigate emotion processing (28, 32, 38, 43, 50, 51).

Neural and behavioral responses to emotional faces differ between healthy individuals and those with various forms of psychopathology, and specific responses to particular emotions (e.g., fear versus anger versus happiness) can differentiate between various forms of psychopathology such as bipolar disorder, depression, anxiety, and aggression (43). In healthy newborns, automatic attention to the eyes occurs very early and is involved in attachment processes and the development of key higher-order processes, such as empathy and theory of mind (25). Researchers have shown that adults with psychopathy as well as adolescents and children with elevated CU traits are hypo-responsive to emotional faces, particularly those displaying fear (41). Further, Adolphs and colleagues (52) have shown that unlike healthy controls, patients with amygdala-damage fail to naturally attend to the most emotionally salient aspects of the environment, such as the eyes of other people, resulting in fear blindness. Other common forms of psychopathology are also characterized by different distortions in emotional attention and recognition (44). For example, individuals with autism, schizophrenia, psychopathy, depression, anxiety, obsessive-compulsive and mania/bipolar disorders all have problems attending to and reading other people's emotions (44). Thus, such impairments can result from neural disorders (53), but can also play a causal role in the development of mental health problems (41). Unfortunately, little is known about the early trajectories of these impairments.

There are a few notable exceptions to this lack of research into the early trajectories of selective attention to critical emotional stimuli, particularly in relation to children with autism and elevated CU traits. There has been mixed evidence relating to whether children with autism spectrum disorder (ASD) show different gaze behaviors than neurotypical children to the eyes and mouths of neutral and emotional faces [e.g., (54–56)]. One study has demonstrated that children with ASD show selective attention to their mother's faces/eyes up until 12 months of age, at which time their attention deteriorates to become a characteristic core feature of the disorder (57). A more recent study with a larger sample size found group differences between toddlers with and without ASD in relation to fixating on faces, but did not replicate the earlier finding of selective attention towards eyes (56). More research is therefore needed to clarify these mixed results. In regards to those with CU traits, a longitudinal study in the United Kingdom (42) tested the current authors' proposal that impairments in selective attention to the caregiver's face is an early marker of CU traits (58). It found that lower selective face tracking at five weeks of age predicted CU traits at 2.5 years old (42). Together, Jones and Klin's (57) and Bedford et al.'s (42) landmark studies highlight the potential of research into the early REAL pathways to reveal critical impairments and timings in the origins of different pathologies that may share similar features, such as difficulties in appropriate empathic response.

While children with ASD and DBD+CU show impaired attention to face/eye features, those with high levels of anxiety are likely to show increased attention to eyes/faces. Adults with anxiety disorders reliably show rapid and unconscious capture of attention by threat stimuli, including human faces and eyes (36). Further, manipulation of this attentional bias reliably reduces anxiety and improves coping (36). There is less research, but clear indications, that similar processes occur in older children (43), yet little research has been conducted examining these processes in early life. Thus, this will be the first study to evaluate attentional capture and preferential tracking of faces and eyes as a predictor of the early emergence of anxiety problems.

#### Responsiveness

Socioemotional responsiveness refers to the gamut of behavioral and physiological responses that a child produces in response to the emotional cues of other people (59). During development, responsiveness is best operationalized using psycho-physiological measures or visceral behavioral responses (29, 30). Visceral behavioral responses indicating arousal include, but are not limited to, approaching or avoiding an unfamiliar toy or person (60–63), reciprocating facial emotional expressions (64), joint attention (31, 65, 66), and a range of behaviors (such as negative reactivity, affect, vocalizations, gaze following, smiling, bids for attention, social reciprocity, etc.) that can be coded from an interaction between an infant and their mother/caregiver (64, 67–73). Psycho-physiological measures that may indicate responsiveness include electrodermal activity (70, 74–76), heart rate variability (70, 76, 77), respiratory sinus arrhythmia (70), vagal tone (67), and pupil dilation (78–81). Emerging research indicates the role of socio-emotional responsiveness in the development of child psychopathology. The circular relationship between socio-emotional attention and responsiveness is purported to further impact on infant's learning and the development of child psychopathology (36). To date, infant socio-emotional responsiveness is primarily understood within the context of a dynamic social interaction, often with the infants' caregiver. The caregiver's role in facilitating and regulating responsiveness may therefore also be an area in which the responsiveness system could be interrupted (70, 71, 82, 83).

#### Learning

Evidence for the association between learning and the development of child psychopathology come from three main areas of research. First, it is well-established that an attentional bias towards aversive facial stimuli (e.g., angry and fear faces) have been linked to the development of anxiety disorders in children (37, 84–86). Second, children with ASD and conduct problems have difficulties with learning from emotional expressions (87). In particular, children with ASD have difficulties with associating the emotional expression of an individual with an object that individual is looking at [e.g., in social referencing, joint attention; (88–90)]. Comparatively, those with conduct problems do not learn to avoid actions that result in facial expressions indicating distress [e.g., sad and fear faces; (91–93)]. Thirdly, research to date has shown that young infants have a proclivity for positive socio-emotional stimuli [e.g., (94, 95)], and that happy faces can be used in evaluative conditioning paradigms with children (96). Although there are few evaluative conditioning studies that have been conducted with infants (32, 96–98), one landmark study has found that infants who focus on faces during conditioning tend to show preferences for selecting stimuli that have been paired with a prosocial emotional facial expression [i.e. happy face; (32)] than an angry emotional face.

Escalating circular relationships between socio-emotional attention, responsiveness, and conditioned learning are also central to the most successful and empirically-supported models of adult anxiety, trauma and depression (36). Similarly, these escalating relationships feature in the emerging models of aggression and antisocial behavior (47, 87, 99, 100). In particular, healthy prosocial behavior involves children learning to avoid aggression as they experience aversive conditioning that results from attending to, and feeling emotional discomfort about, other people's negative emotions (87). Within the REAL model, aggressive antisocial behavior represents a failure of this developmental process whereby the child fails to learn competent social cognition, empathy, and to inhibit aggressive antisocial behavior, due to impairments in emotional attention, responsiveness, and the learning that results.

### Objective of the Proposed Study

In this paper, the protocol for the longitudinal *“Watch Me Grow for REAL”* study is presented. In this study, the specific pathways of early-onset mental health disorders are examined using a prospective birth cohort. The primary aim of this research is to map the emergence of the REAL variables through the first three years of life to predict the emergence of the major childhood mental health problems, as well as social adjustment and impairment more generally. Specifically, the growth of the neurodevelopmental systems associated with the REAL constructs will be modeled over the early childhood years, as they interact with the quality of the environment/caregiver interactions to predict the emergence of psychiatric illness, represented by the DBD (+/− CU), anxiety disorders, ADHD, and ASD, as well as social adjustment and impairment. These hypotheses reflect a key assumption of the REAL model that environmental influences are not unimportant to the development of mental health problems, just that they are non-specific. As such, no differences across disorders are predicted for these contextual influences and, instead, differences in the risk for the various psychiatric illnesses are proposed to be due to variation in the REAL constructs.

### Hypotheses

The original hypotheses for the REAL model as outlined by Dadds and Frick (23), are presented in **Table 1**. Of these, the REAL hypotheses that will be specifically examined in this longitudinal birth cohort study are:

**Table 1:** Original hypotheses about the REAL constructs that identify the common and unique features of emerging developmental psychopathology as outlined by Dadds and Frick (23), where + is elevated and − is deficit.

Disorders	Socio-emotional Attention	Non-emotional Attention	Socio-emotional Responsiveness	Non-emotional Responsiveness	Socio-emotional Learning	Non-emotional Learning
DBD-CU	+		+		+	
Anxiety	+	+	+	+	+	+
DBD+CU	–		–		–	
ASD	–	+	–		–	
ADHD		–

1. Socio-emotional attention is expected to be elevated for children who are shown to develop DBD-CU and anxiety disorders, and is expected to be deficient for children who are shown to have DBD+CU traits and ASD.
2. Socio-emotional responsiveness is expected to be elevated for children who are shown to develop DBD-CU and anxiety disorders, and is expected to be in deficit for children who are shown to have DBD+CU and ASD.
3. Socio-emotional learning is expected to be elevated for children who are shown to develop DBD-CU and anxiety disorders, and is expected to be in deficit for children who are shown to have DBD+CU and ASD.
4. In regards to differentiating between the risk profiles for DBD-CU and anxiety disorders, it is expected that children who are shown to have anxiety disorders will demonstrate elevated attention, responsiveness, and learning to non-emotional stimuli (such as in behavioral inhibition tasks) in comparison to children who are shown to have DBD-CU.
5. In the case of ASD, it is also expected that REAL impairments will be observed with regard to the self as relational object. In particular, when compared to non-ASD children, ASD children will show impairments in orienting their attention to “self” stimuli (e.g., orienting to their name) and in initiating joint attention.

Although ADHD is mentioned in this protocol paper, it should be noted that ADHD is proposed to not be characterized by core disturbances in the REAL variables (23). However, ADHD is a disorder marked by poor attentional control in general, and it co-occurs at a high rate in children with DBD (+/- CU), ASD, and anxiety problems. Thus, it is crucial to include ADHD in the models to control for general disturbances in attention, against which specific disturbances in emotional attention can be compared.

## Methods and Analysis

### Design

This study has a longitudinal design following a prospective birth cohort. Data will be collected from four assessments and mothers' electronic medical records. Data from mothers' electronic medical records will include demographic information, psychosocial and health data collected during mothers' 20-week antenatal midwife appointment, health data from birth records, and health data from Community Health Nurse home visits. Mothers will complete a Newborn Baseline Questionnaire within a week of the birth of their child (at the time of recruitment). Mothers and children will then attend laboratory visits when the child is aged between 6- and 11-months-old (Time point 1; T1), 12- and 23-months-old (Time point 2; T2), 24- and 35-months-old (Time point 3; T3), and 36- and 47-months-old (Time point 4; T4). During each laboratory visit, mothers will complete questionnaire and behavioral measures, and children will complete eye-tracking and behavioral tasks to assess the REAL variables and contextual variables. Mothers will be reimbursed with a $40 gift card voucher following each laboratory visit to thank them for their time and to contribute towards their transportation costs. Fathers of children enrolled in the study will be invited to participate by completing questionnaire measures, starting from T1. The University of Sydney Human Research Ethics Committee (Project Number 2017/644), and the South Western Sydney Local Health District Human Research Ethics Committee (Local Project No HE17/115) approved this study. Stakeholder consultation was not conducted for this study.

### Selection of Participants

Mothers that are eligible to participate will be those who have given birth between October 2017 and December 2018 at Liverpool Hospital in the South Western Sydney Local Health District in Sydney, NSW Australia. The South Western Sydney Local Health District is the largest health service in New South Wales in Australia, and has been found to be broadly representative of the socially disadvantaged and culturally diverse Australian population (101).

The inclusion criteria for this study are having a child born at Liverpool hospital during the recruitment period, maternal intention to remain in Sydney for four years, and maternal functional English (or a family member willing, and able, to translate throughout the project). The exclusion criteria for this study are children who are known to have congenital conditions at birth. Mothers will also be excluded from the study if nursing staff report to the research team that the mother will not be taking her child home from the hospital (e.g., infant death, forced removal by child services, etc.). Fathers will be eligible to participate in the questionnaires if the mother of their child has enrolled in the study, and the child's mother is willing to provide the father's contact details.

Mothers who do not meet exclusion criteria will be approached during their stay in the postnatal ward at Liverpool Hospital by the researchers. The researchers will attend the postnatal ward of Liverpool Hospital 1-2 times each weekday during the recruitment period to explain the study to mothers who had recently given birth. They would give mothers (along with their partners, if available) information about the study. If mothers indicated interest in taking part in the study, they will be provided with the study's information statement, written consent form, and Newborn Baseline Questionnaire to complete. Completed forms could be handed back to the researchers, placed in a locked mailbox located in the postnatal ward, or posted to the research team.

One longitudinal study has been conducted in the South Western Sydney Local Health District using the same recruitment strategy (101). In this study, the proportion of infants with the following factors for future developmental risk were examined: perinatal risk (low birth weight, and/or preterm and/or admission to the Special Care Nursery/Neonatal Intensive Care Unit); maternal Middle Eastern or Asian nationality; English not being the primary household language; and/or neighbourhood Socio-Economic Indexes for Areas (SEIFA) score in the lowest decile. It was found that 35% of infants recruited for this previous study were exposed to one risk factor, 23% were exposed to two, 14% were exposed to three, and 2% were exposed to four risk factors (101). Thus, the population from which our sample will be recruited is ideal for over-sampling infants who have been exposed to developmental risk factors.

### Measures and Methods

#### Antenatal Data

As part of routine antenatal care, mothers will complete an interview with a midwife at approximately 20 weeks gestation in which they will report demographic details, psychosocial information (e.g., their response to pregnancy, mental health history, childhood abuse history, etc.), medical history (e.g., parity, alcohol use during pregnancy, smoking during pregnancy, drug use during pregnancy, etc.), and will respond to the Edinburgh Depression scale (102, 103). The total score from the Edinburgh Depression scale will indicate mothers' depression symptomatology during the past week, with higher scores indicating greater depressive symptoms.

#### Birth Data

Routinely collected birth data will be recorded from mothers' electronic medical records. These data will include the child's date of birth, sex, weight, length, head circumference, birth type, obstetric and maternal complications, and Appearance, Pulse, Grimace, Activity, and Respiration (APGAR) scores.

#### Newborn Baseline Questionnaire (NBQ)

Mothers will self-report on the NBQ at the time of recruitment (i.e. within one week of the child's birth). Mothers will be asked to respond to demographic items (e.g., marital status, household income, parents' ethnicity, parents' education level, etc.), current breastfeeding status and intentions, perceived social support, perceived satisfaction with household income, perceived satisfaction with neighborhood conditions, family history of mental health concerns, family history of drug/alcohol problems, family history of learning-related problems, and medical history (e.g., alcohol use during pregnancy, smoking during pregnancy, drug use during pregnancy, physical health problems etc.).

##### Overall Relationship Satisfaction

As part of the NBQ, mothers will be asked to respond to the Overall Relationship Satisfaction item from the Dyadic Adjustment scale (104) to indicate how happy they are with their relationship with the baby's biological father. Mothers will be asked to rate the happiness in their relationship on a 7-point scale ranging from 0, *extremely unhappy*, to 6, *perfect*.

##### Satisfaction With Childbirth

Mothers will also be asked to respond on the NBQ to the seven-item Satisfaction With Childbirth scale [SWCh; (105)] which will examine mothers satisfaction with their most recent birthing experience. Mothers will be asked to rate the SWCh items on a 7-point Likert scale ranging from 7, *strongly agree*, to 1, *strongly disagree*.

#### Postnatal Data

Routinely collected health data will be extracted from mothers' electronic medical records. These data will include mothers' Edinburgh Postnatal Depression scores (103) that will be collected by Community Health Nurses at postnatal home visits. These visits typically occur within one month of hospital discharge.

#### REAL Measures

**Table 2** shows a summary of the REAL constructs being examined in this study and their associated measures.

**Table 2:** REAL constructs being examined in the Watch Me Grow for REAL study and the associated measures.

REAL Construct	Measures
Attention	Modified attention disengagement task; Infant dot-probe task; Joint attention task; Social orienting to socio-emotional stimuli and non-emotional stimuli; Still-Face paradigm: behavioral coding; Behavioral inhibition; Free play: behavioral coding.
Responsiveness	Joint attention task; Eye-tracking tasks: pupil dilation Still-Face Paradigm: Electro-dermal activity (EDA), child heart rate coded from facial skin color; and behavioral coding; Behavioral inhibition: EDA, child heart rate coded from facial skin color; and behavioral coding; Free play: EDA, child heart rate coded from facial skin color; and behavioral coding.
Learning	Evaluative conditioning task; Behavioral inhibition.

##### Eye-Tracking Tasks

Laboratory sessions will include up to four eye-tracking tasks, as described below. Eye-tracking data will be collected using a Tobii Pro X2-60 eye tracker (Tobii Technology, Stockholm, Sweden) that samples data at a rate of 60Hz. This eye tracker will be attached below the computer monitor. Tasks will be programmed using MATLAB and presented on a computer monitor with 1920 x 1080 pixels. Participants will be positioned approximately 60cm to 75cm from the computer screen. Wherever possible, children will be seated on their mother's laps at T1 and T2 visits, and in a child seat at T3 and T4. Each testing procedure will begin with a five-point calibration procedure using looming multi-colored circles. Fixations, areas of interest, and pupil dilation data will be collected across tasks.

###### Modified Attention Disengagement Task

**Figure 2:**
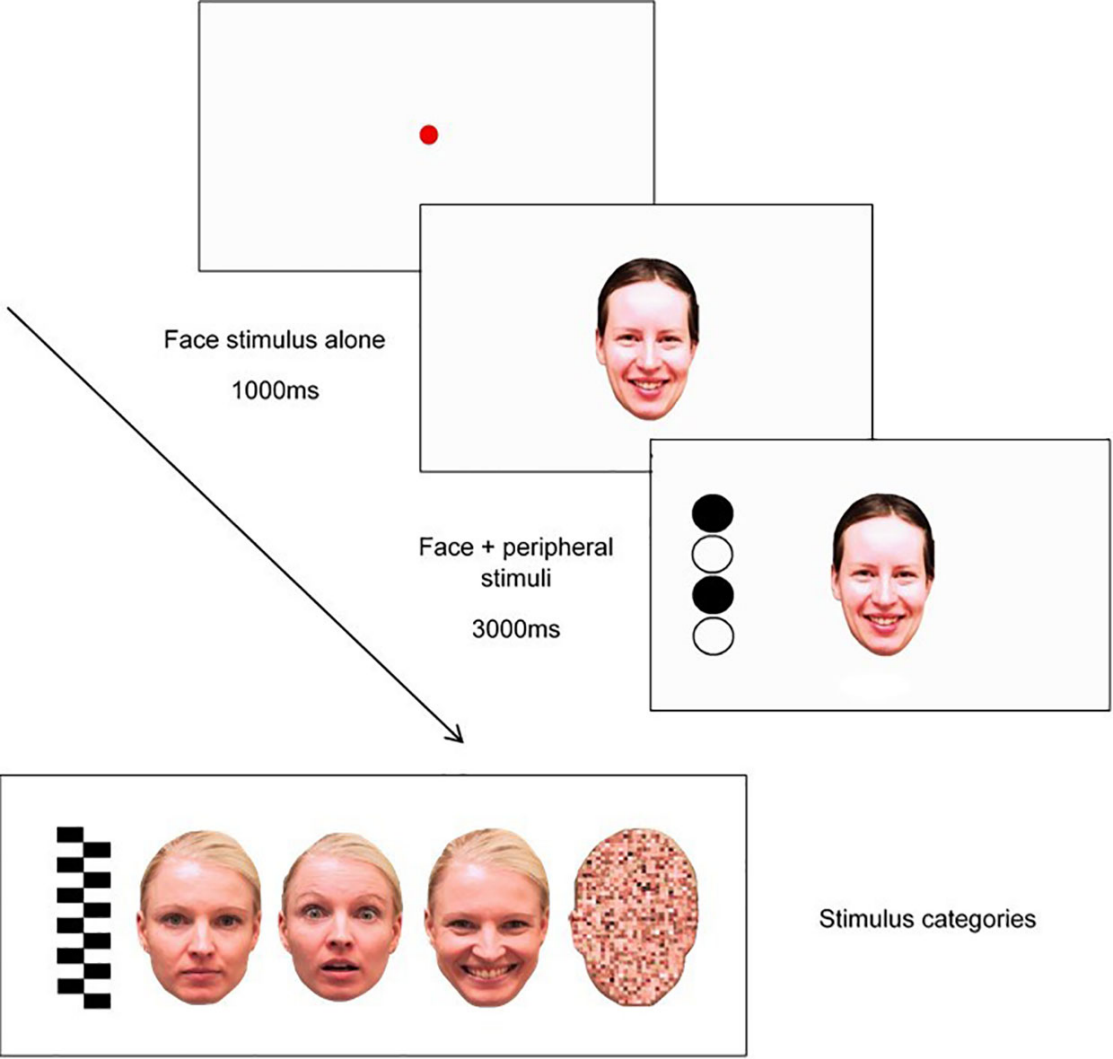
Trial sequence used in modified attention disengagement task, including examples of stimulus categories (pictured L-R: peripheral stimulus (**“**target**”**), neutral face, fearful face, happy face, and scrambled face).

This task will be based on Peltola, Leppänen (50), and will measure attentional disengagement from static socio-emotional facial expressions and a non-emotional pixilated control image using eye gaze. This task will be completed at T1, T2, T3, and T4. The sequence of this task will involve three elements: a central face stimulus, a peripheral “target” stimulus, and a fixation point, as shown in **Figure 2**.

Each trial will begin with a fixation point, depicted as a red circle, that expands and contracts repeatedly to capture the child's attention (from 0.4° to 4.3°). The gaze criterion for fixation stimulus will be 100 ms. Fixation will be followed by a presentation of one of eight central stimuli (i.e., one of two versions of either a neutral face, happy face, fearful face, or a pixilated non-emotional scrambled image in the shape of a face) for 1000ms in the center of the screen where the fixation point had been located. As shown in **Figure 2**, faces will measure 15° and 11° vertically and horizontally, respectively. After the 1000ms face stimuli presentation, a target stimulus will appear on one side of the screen at 14° distance of the face. Target stimuli (measuring 15° × 4° visual angle) will either be a set of black-and-white vertically arranged circles or a checkerboard pattern. The side on which the target appears (left or right side of the emotional/scrambled face stimuli) will be randomized. Each trial will end after 3000ms have lapsed, with an inter-trial interval of 1000ms. Each of the eight face stimuli (i.e., neutral, happy, fearful, scrambled) will appear six times in randomized order (i.e., for a total of 48 trials); however, the same central stimulus type will be presented no more than four times in a row, and the side on which the target appears (left/right) will be repeated no more than four times in a row.

###### Infant Dot-Probe Task

**Figure 3:**
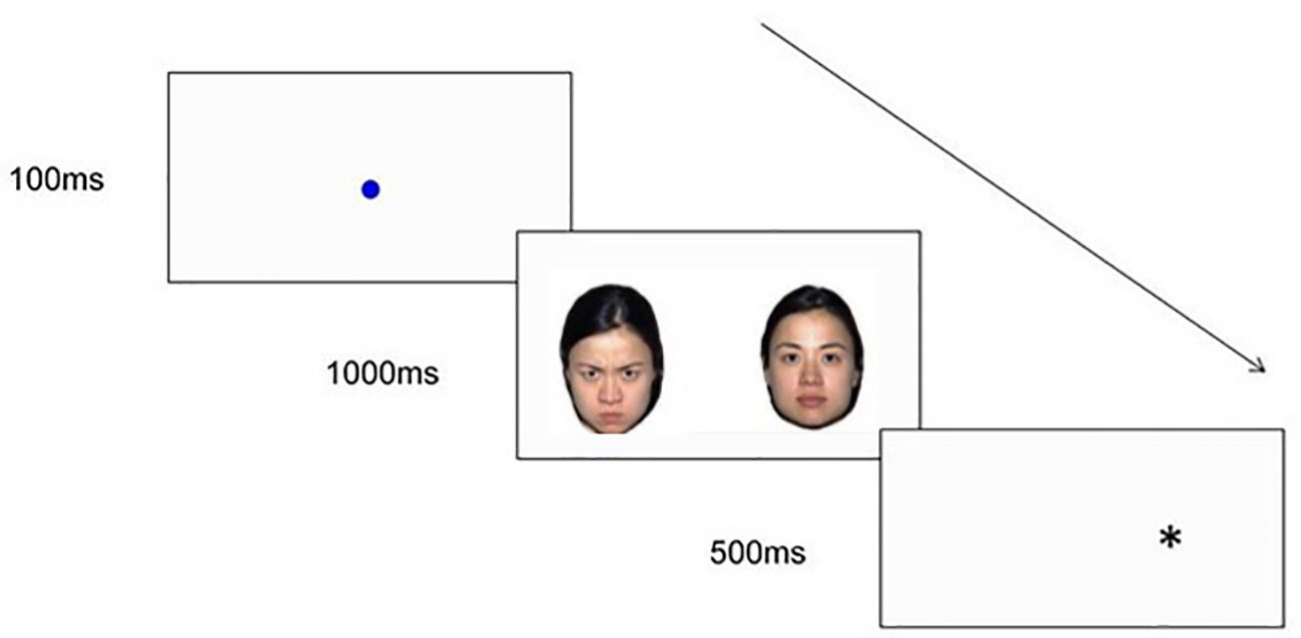
An example of an Angry-Neutral incongruent trial sequence used in the infant dot-probe task.

The infant dot-probe task will be an infant version of the dot-probe task that is widely used in the child and adult literature to measure spatial attention to emotional stimuli. The infant dot-probe task used in this study will be based on Pérez-Edgar, Morales (28) and will measure infant's attentional orienting to emotional face stimuli. This task will be completed at T1, T2, and T3. This task will consist of 30 experimental trials. Each trial will begin with a fixation stimulus (a looming blue circle), which will be presented until the infant fixates continuously for at least 100ms (28). As depicted in **Figure 3**, fixation will be followed by the presentation of one of three types of face pairs, drawn from the NimStim face stimulus set (106): Happy-Neutral (six congruent trials, six incongruent trials), Angry-Neutral (six congruent trials, six incongruent trials), and Neutral-Neutral (six trials), with three male and three female models for each emotion. Face pairs will be followed by the presentation of a dot probe in one of the two face stimulus locations. Trials will be considered congruent if the probe appears in the same location as the emotional face (i.e. angry or happy face; see **Figure 3**), and incongruent if the probe appears in the opposite location to the emotional face.

###### Joint Attention Task

Children will complete Billeci and colleagues' (31) eye-tracking joint attention (JA) task at T2, T3, and T4. This task will measure socio-emotional attention. The gaze criteria for the looming ball will be set at 200ms. This task will consist of three types of trials, measuring responding JA, and initiating JA to both predictable and unpredictable events. There are three components of each trial: the model looking down (2 s); the model holding a direct gaze towards the camera, smiling, and saying, “Ciao!” (2 s); and finally, the JA scene (4 s for responding JA, and 7 s for initiating JA). In a responding JA trial, the model will turn her head towards one of the two objects in the scene and will fixate on this object (target object). In an initiating JA to predictable events trial, one of two toy cars in the scene will move to the center of the screen (target object), while the model will maintain a direct gaze towards the camera and neutral expression. In an initiating JA to unpredictable events trial, a toy truck (target object) will appear from outside the scene and will move from one side of the screen to the other, while the model maintains the direct, impassive gaze. The order of the trials will be organized into two blocks depending on whether the target object is on the right or the left side of the scene. There will be two versions of each trial type, such that there will be six trials in total. Both the order of the blocks and the order of the trial types within these blocks will be randomized. Outcome measures for this task will be as per Billeci, Narzisi (31) and include reaction times between areas of interest (e.g., face to target object), and differences in reaction times across different trials (e.g., difference between frequency of first looks to target moving object and frequency of first looks to non-target still object).

###### Evaluative Conditioning Task

**Figure 4:**
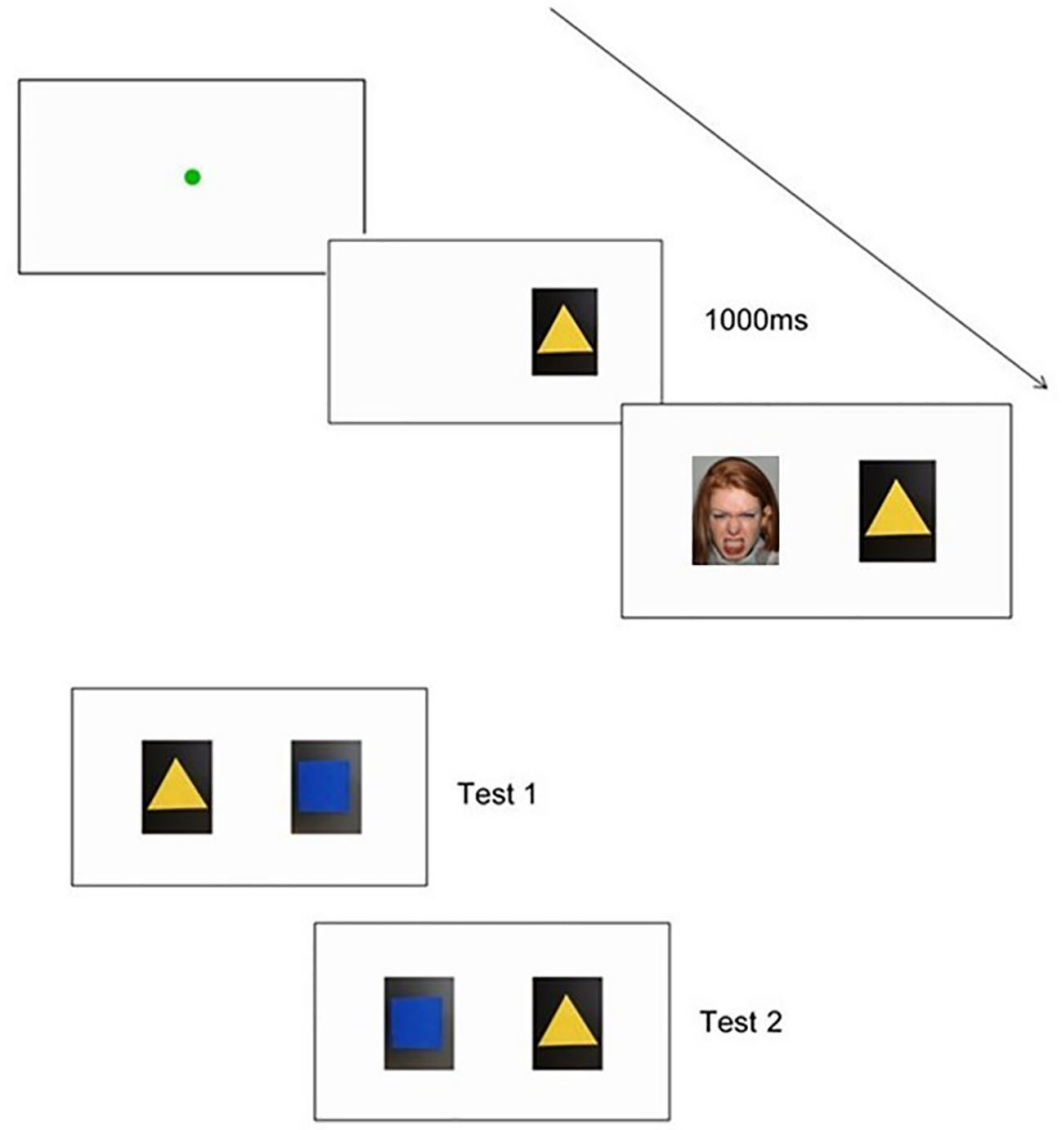
An example of a trial sequence used in the Evaluative Conditioning Task, including a shape and face pairing. Also pictured are the examples of both choice test presentations.

For the evaluative conditioning task, changes in infants' preferences for neutral shapes will be measured after these shapes are repeatedly paired with different socio-emotional faces. This task will be pilot tested at T1, and run with the full sample at T2 and T3. In accordance with Richmond, Zhao (32), the socio-emotional stimuli will be neutral colored shapes that are paired with static happy or angry faces. The task will begin with a fixation point (green circle expanding and contracting repeatedly) to capture the child's attention, from 0.4° to 4.3° preceding each trial. The task will commence once the child is looking at the fixation point for at least 100ms and once the fixation point is at its smallest. During the conditioning phase, children will be shown pairings of one shape paired with happy faces, and another shape paired with angry faces. In line with Richmond, Zhao (32), the neutral colored shape will be presented on one side of the screen for 1 s before the face appears with it on the other side of the screen, to ensure that children fixate on both the object and the face. The face and shape stimuli will appear on either side of the fixation point ten centimeters apart. Each image will measure 180 x 230 pixels and will appear at 11.1 x 12.4 centimeters on the screen. The side on which the face/shape appears (left, right) will be randomized but constrained to no more than four times in a row on the same side. The stimuli will be displayed until the child looks away from the screen. Once the child looks away from the screen, the red loomer fixation point will appear again to draw their attention back to the screen, before presenting the next trial. Object pairings will continue until habituation occurs, or if the examiner determines that the infant is becoming too fussy or fatigued to complete any more training test trials. Object pairings will be counterbalanced across children (32). Two outcome measures will be used to determine whether conditioning has occurred (32): (1) looking preferences to the two shapes when shown side by side on the computer monitor (across two 10-s trials), and (2) behavioral choice by examining the object that the infant appeared to prefer (either first touch or longer duration of touch). Children's behavior will also be videotaped. To determine inter-rater reliability, an independent coder will code a random subset of participants' videotaped recordings at each time point. **Figure 4** illustrates the sequence of the task.

##### Behavioral and Interaction Tasks

###### Social Orienting to Socio-Emotional Stimuli and Non-Emotional Stimuli

This task will be based on Dawson et al.'s (26, 27) social orienting task, and will use some of the same socio-emotional and non-emotional stimuli as presented in Dawson, Toth (26). In particular, the two live socio-emotional stimuli will be: (a) humming, and (b) calling the child's name. The two non-emotional stimuli will be mechanical sounds of inanimate objects: (c) a timer beeping, and (d) a toy phone making a noise. The examiner will assume a neutral facial expression when delivering the socio-emotional stimuli and will turn their head towards the floor when delivering the non-emotional stimuli to ensure that the child's response is not unduly influenced by personal or social characteristics specific to the examiner. Stimulus order and the location of stimuli presentation will be counterbalanced across participants. This task will be completed at T1, T2, and T3. At T1 and T2, the infant will be seated on his/her mother's lap. At T3, the child will be seated on a small chair at a small table. The child will be given a mildly interesting toy to play with, and once the child is engaged in play and not looking at the examiner, the examiner will deliver the stimuli. Each stimulus will be delivered three times with a 1-s interval between trials, for a total presentation time of approximately 6-s per stimulus. In accordance with Dawson et al. ((26), pp. 275), orienting in this task will be defined as “turning the head and/or eyes toward an auditory stimulus. If a child turned his or her eyes and/or head toward the stimulus, the response was coded as a correct response whether or not the response included shared attention with the examiner.” For each stimulus the examiner will record at which trial the child first oriented to the stimulus (i.e. 1, 2, or 3). If the infant fails to turn his or her head or eyes toward the stimulus on any trial, the examiner will record this as a lack of response. Therefore, an overall lack of responsiveness will be indicated by a failure to attend to any of the stimuli. Children's behavior will also be videotaped. To determine inter-rater reliability, an independent coder will code a random subset of participants' videotaped recordings at each time point.

###### Face-to-Face Still-Face Paradigm

Attention to the mother's face will be assessed during the Face-to-Face Still-Face Paradigm (107). In the Still-Face Paradigm, infants will typically find their mother's neutral face to be aversive, at first responding by making bids to re-engage the mother, and when this fails showing less positive affect, more negative affect, and gaze aversion (64, 108). There will be three face-to-face interaction episodes in this task: baseline face-to-face play (in this study, four minutes) where the mother will engage in “normal” play facing their infant; still-face episode (90 s) where the mother will become unresponsive and maintain a neutral facial expression; and a “reunion” face-to-face episode where the mother will resume play with the infant. The Still-Face Paradigm will be conducted in a small room fitted with cameras to video record the mother and child. This task will be completed at T1, T2, and T3. At T1, the infant will be placed in a bouncing chair, and the reunion play will be conducted for four minutes. At T2, the infant will be placed in a high-chair, and the reunion play will be conducted for two minutes. At T2, experimenters will initiate each episode by knocking on a one-way mirror.

At T3, the Toddler Still-Face Paradigm (109) will be used. The procedure from Weinberg, Beeghly (109) will be adapted so that the commencement of mothers' unresponsive Still-Face expression will occur either: the first time that the child attempts to engage with the mother following their four minutes of free play, or if the child does not attempt to look at/engage with the mother following their four minutes of free play then after another two minutes elapses, such that the first free play session will last for no longer than six minutes. This change was made as it has been found that the timing of the break in the interaction tends to be more pronounced when the Still-Face occurs when an infant is preparing for an interaction with his/her mother than when the Still-Face occurs after a natural “end point” of interaction (64). The unresponsive Still-Face will be conducted for 90 s, and the reunion play will be conducted for two minutes. At T3, experimenters will initiate each episode by knocking on a one-way mirror.

During the Still-Face Paradigm, skin conductance will be measured using EDA. Two EDA sensors will be placed on the soles of children's feet and will record children's physiological response during the different episodes of the task. Socks will be placed over the EDA sensors and children's feet. Further, Baby FaceReader in Noldus will be used to code child heart rate using children's facial skin color.

###### Child Behavioral Inhibition

Behavioral inhibition will be examined by observing children's approach or avoidance behaviors towards novel non-emotional stimuli (110). At T2, T3, and T4, children will be presented with a novel robot that moves/dances whilst in the presence of their mother. Researchers will use a different robot at each time point to maintain the novelty of the stimulus. Similar to Fox, Henderson (61) and Aktar and colleagues (63, 111), infant's proximity to their mother, latency to touch the robot, and latency to make a fear response will be coded from video-tapes. In addition, Baby FaceReader in Noldus will be used to code child heart rate using children's facial skin color.

###### Free Play

At T2, T3, and T4, mothers and infants will be provided with age-appropriate toys and asked to play as they would at home for four minutes. At T4, a pack up component will be added to this task for 1 minute following the four minute free play. Free play interactions and the pack up task will be videotaped for later coding, including using Baby FaceReader in Noldus.

#### Contextual Variables

Perceived social support, perceived satisfaction with household income, perceived satisfaction of neighborhood conditions, and several demographic items (e.g., marital status, household income, current education level, etc.) will be re-assessed at T1, T2, T3, and T4.

##### Overall Relationship Satisfaction

Parents will be asked to report their Overall Relationship Satisfaction (104) at T1, T2, T3, and T4 with the child's other biological parent and, if relevant, their current romantic partner. Parents will be asked to rate their happiness in these relationships on a 7-point scale ranging from 0, *extremely unhappy*, to 6, *perfect*.

##### Parent-Child Bonding

At T1 only, mothers and fathers will be asked to complete the Postpartum Bonding Instrument (112, 113) to examine their bonding with their child. Parents will be asked to report on a six-point scale, ranging from 0, *never*, to 5, *always*. Although this scale was initially developed for use with mothers, in this study it will be extended to also examine fathers' bonding with their child.

##### Parent and Child Adverse Life Experiences

Mothers and fathers will be asked to report adverse life experiences for themselves, as well as for their child on the Adverse Life Experiences Scale [ALES; (114)]. If parents report that an adverse life experience has occurred for themselves or their child, they are also asked to indicate the age(s) when the experience(s) occurred. Mothers will be asked to report on their own adverse life experiences at T1, and fathers will be asked to report on their own adverse life experiences at T2. Mothers and fathers will be asked to report on their child's adverse life experiences at T4.

##### Parental Mental Health

Mothers and fathers will report on their own mental health during the past week using the 21-item Depression Anxiety Stress Scales [DASS21; (115)] at T1-T4. Parents will respond to items on a four-point scale, ranging from 0, *did not apply to me at all*, to 3, *applied to me very much, or most of the time*.

##### Parenting Practices

Mothers and fathers will report on their own parenting practices (consistency, coercive parenting, positive encouragement), their parent-child relationship, their own adjustment, family relationships, and (if they are in a romantic relationship) parental teamwork during the past four weeks by responding to the Parenting and Family Adjustment Scales [PAFAS; (116)]. Only age-appropriate items will be asked at each assessment (i.e. only items 14-30 at T1, but all items at T2-T4). Parents will respond to items on a four-point scale, ranging from 0, *not true of me at all*, to 3, *true of me very much, or most of the time*.

##### Child Temperament

At T1, mothers and fathers will report on child temperament using the Infant Behavior Questionnaire Revised - Very Short Form (117). At T2, mothers and fathers will report child temperament on the Infant Behavior Questionnaire - Extremely Short REAL version. At T3 and T4, mothers and fathers will report child temperament on the Early Childhood Behavior Questionnaire - Extremely Short REAL version. The two Extremely Short REAL questionnaires were developed for this study following the approach from Sleddens, Kremers (118), and each measure consists of three items with one item tapping into surgency, one item tapping into negative affectivity, and one item for effortful control. For both scales, parents will be asked to consider how true each statement is for their child, by rating it on a five-point scale, 0 = *extremely untrue of your child*, 1 = *slightly true of your child*, 2 = *partially true/partially untrue of your child*, 3 = *quite true of your child*, and 4 = *extremely true of your child*.

##### Child Development

At T1-T4, parents will report on their child's development on the age-appropriate version of the Ages and Stages Questionnaire 3 [ASQ-3; (119)]. The ASQ-3 assesses child development across five domains: Communication, Gross Motor, Fine Motor, Problem Solving, and Personal-Social.

##### Stool Samples and Mother-Child Intake

Emerging research indicates there may be a link between human microbiome and mental health (120–122). In the current study, an exploration of the biological vulnerabilities to experience (23) will be examined through the collection of infant stool samples at T1. Mothers will also be asked to report at T1 on their child's diet, as well as the mother and child's history of antibiotic use.

#### Outcome Variables

##### Social and Emotional Well-Being

###### Brief Social and Emotional Well-Being Questionnaire

At T1, T2, and T3, mothers and fathers will complete a short social and emotional wellbeing measure that is being assessed as part of this study to assess child mental health symptoms. Four items will measure child socio-emotional dysfunction with one item from the Antisocial Process Screening Device (APSD) – Callous Unemotional sub-scale (123), one item from the Inventory of Callous–Unemotional Traits (124), one item from the Modified Checklist for Autism in Toddlers[M-CHAT; (125)], and one item from the Social Responsiveness Scale 2 – preschool form (SRS-2; (126)]. Child anxiety symptoms will be measured using three items from the Revised Preschool Anxiety Scale [PAS-R; (127)]. Child control and aggression will be measured using five items, with four items from the Cardiff Infant Contentiousness Scale (128) and one item from the Strengths and Difficulties Questionnaire [SDQ; (129)]. Parents will respond on a five-point scale, ranging from 0, *never*, to 4, *all of the time*. The psychometric properties of this brief measure have not been published, and require further validation with other clinical and non-clinical cohorts.

###### Strengths and Difficulties Questionnaire

At T4, mothers and fathers will provide an indication of their child's social-emotional and behavioral adjustment using the 2- to 4-year-old version of the SDQ (129). The total difficulties score will be used as general measure of child social adjustment/impairment. Parents will respond on a three-point scale, ranging from 0, *not true*, to 2, *certainly true*.

##### Disruptive Behaviors

At T4, the conduct problems subscale of the 2- to 4-year-old version of the SDQ (129) will be used to measure children's disruptive behaviors. At T3 and T4, disruptive behaviors will also be measured with items from Stringaris and Goodman (130) that have been previously used to index specific dimensions of oppositionality. This questionnaire will include 11 items that are rated on a three-point scale from 0, *not true*, to 2, *certainly true*; and three items that will be rated from 0, *never*, to 2, *often*.

##### CU Traits

At T4, mothers and fathers will report on their child's CU traits on the 24-item parent-report preschool version of the Inventory of Callous-Unemotional Traits (131). Parents will report on a four-point scale, ranging from 0, *not at all true*, to 3, *definitely true*.

##### Autism Symptoms

###### Modified Checklist for Autism in Toddlers

At T3, mothers and fathers will report child autism symptoms on the 20-item Modified Checklist for Autism in Toddlers [M-CHAT; (125)]. For each item, parents will respond either *no*, 0, or *yes*, 1.

###### Autism Spectrum Quotient

At T4, mothers and fathers will report child autism symptoms on the child version of the Autism Spectrum Quotient (132). Parents will respond to 10-items on a four-point scale, ranging from 1, *definitely disagree*, to 4, *definitely disagree*.

##### Anxiety Symptoms

At T4, mothers and fathers will report child anxiety symptoms on the 28-item Revised Preschool Anxiety Scale [PAS-R; (127)]. Parents will respond on a five-point scale, ranging from 0, *not true at all*, to 4, *very often true*.

##### ADHD Symptoms

At T4, mothers and fathers will report ADHD symptoms on the ADHD Rating Scales IV – Preschool version (133). Parents will respond on a four-point scale, ranging from 0, *never or rarely*, to 3, *very often*.

##### Child Mental Health Diagnoses

For those children scoring above the cut-off score on the SDQ or any of the parent-reported symptoms measured at T4, diagnostic interviews will be conducted. The Diagnostic Interview Schedule for Children (DISCAP) will be used to assess for DSM-5 diagnoses for child mental health disorders (134).

#### Data Analysis

The core design yields a set of repeated measures of the REAL constructs, and outcome indices of psychopathology. Latent growth variables for each of the REAL constructs will be developed for the longitudinal growth models. This produces a measurement model and starting points (intercept) and slopes for each measure and is important to ensure continuity of common variance of constructs over age changes. The outcomes variables will be used categorically as diagnoses, whereby category membership is the criterion variable and risk odds ratios are parsed into common variance (any psychopathology) and unique membership (such as the presence of ASD without concurrent diagnosis), and as continuous dimensional variables. Latent profile analyses will also be run to examine whether there is consistency in latent profiles across tasks relating to the REAL constructs. Further, the predictors and correlates of class membership will be examined. Latent transition analyses will also examine whether the group of individuals in each latent class based on REAL variables changes at each time point. Missing data will be examined for non-random patterns, and appropriate missing data strategies will be used to model any trends found. All analyses will be conducted using the contextual variables of environmental adversity and developmental progress/delay as covariates.

#### Sample Size

**Figure 5:**
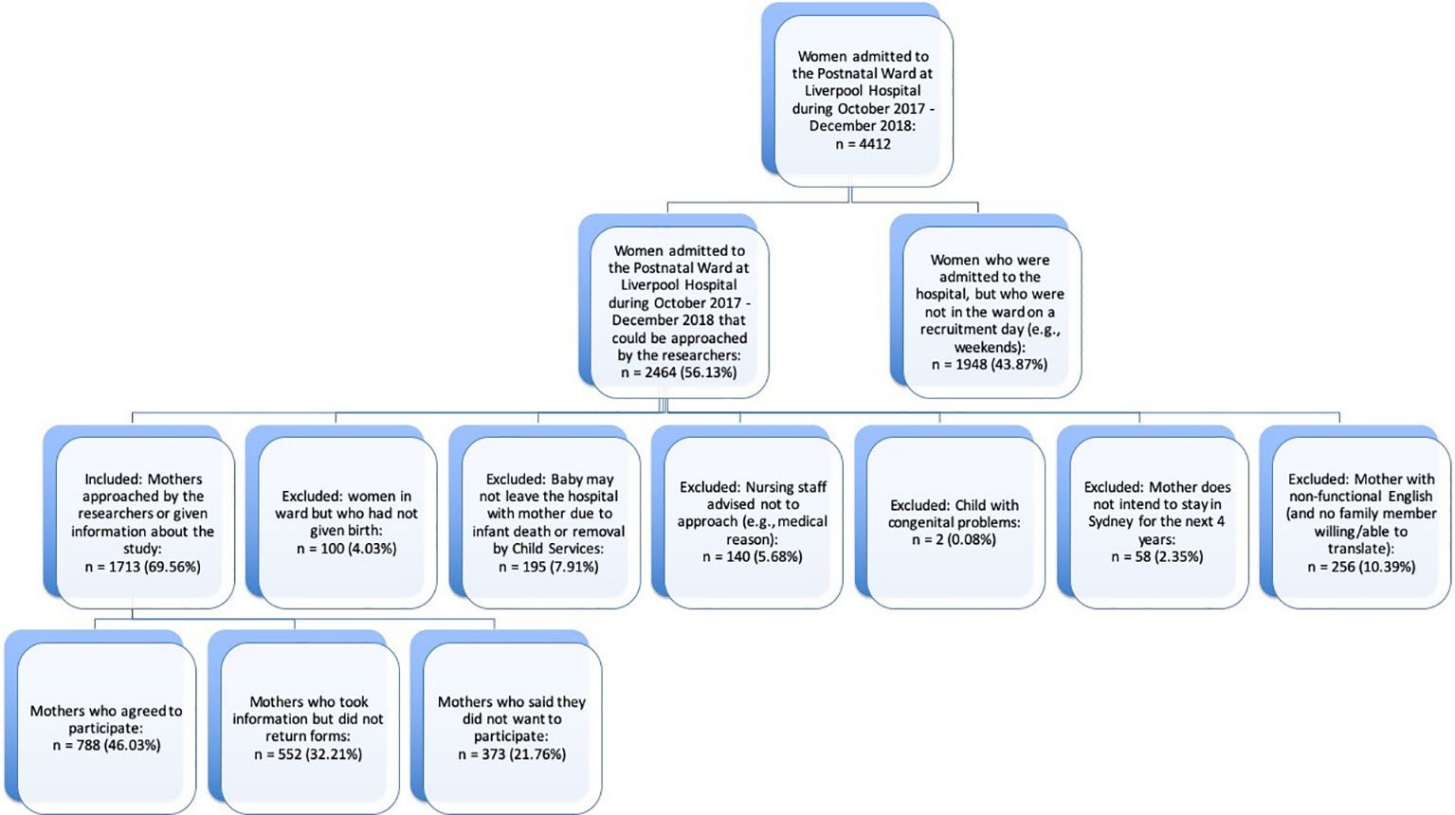
Recruitment CONSORT Diagram.

Longitudinal designs and growth modeling are notoriously difficult to assess for statistical power. The first consideration is for categorical analyses where power is generally the lowest as they split the sample into subgroups. We present power analyses in two ways. For predicting dimensions of psychopathology at T4 using regression, G*Power v3.1.9.2 shows the effect size detectable at power = 0.8, error = 0.05, 3 main predictors and 10 control variables, and a sample size of *N* = 450 shows we have the sensitivity to detect a small effect size of f2 =.024. For cross sectional categorical comparisons of psychopathology at the T4 assessment, the lowest power is available to test for ASD given it has the lowest expected base rate. Using G-power for Chi-squared tests with power = 0.8, significance at p=0.05, and a small effect size of w = 0.02, we require a total sample size of *N* = 321. Thus, the study is powered to detect the both categorical and dimensional tests of emerging psychopathology. Our final sample is *N* = 788 children (see **Figure 5**). Using the best available Australian and United States of America-European epidemiological studies to estimate the following numbers of children, it is estimated that the following full and at-risk diagnoses/profiles will be observed in the sample at T4: DBD-CU – full 10%, *n* = 79; at-risk 25%, *n* = 197); DBD+CU – full 6% *n* = 47, at-risk 15% *n* = 118; anxiety disorders – full 10% *n* = 79, at-risk 25% *n* = 197; ASD – full 1.5% *n* = 12, at-risk 8% *n* = 63. These are overlapping, non-mutually exclusive groups.

## Discussion

In the model presented in this paper, it is argued that emotional attention, responsiveness, and learning (REAL) have the potential to explain common and unique variance in the early presentation of the common child psychopathologies of DBD-CU, DBD+CU, anxiety, ASD and ADHD. This study will be the first to test a clear developmental map of the REAL constructs and identify both the unique and specific causes of childhood psychopathology. The idea that REAL deficits are trans-diagnostic is consistent with the growing recognition that psychiatric disorders are not distinct categories but overlapping neurodevelopmental disorders (18). Healthy humans are born with exquisitely-tuned neural architecture for recognizing and attending to faces and eyes, and this underlies development of higher socio-emotional skills, conscience, theory of mind, empathy, and aspects of language and cognition (25, 34).

Early identification of impairments in this architecture can have clear and powerful implications for early assessment and treatment. For example, with regard to DBD, experimental manipulations of attention, whereby children with high CU traits and adults with psychopathic traits are trained to increase attention to emotional cues, are reliably associated with improved recognition, emotion, and learning in the presence of salient emotional stimuli (99, 135). Training attention away from emotional cues reduces fear in experimental manipulations and promotes positive coping in people with high anxiety and related disorders (36). In autism and DBD, training attention to the eyes can improve emotion recognition (136). These findings illustrate the potential treatment implications for the REAL constructs. It must be noted, however, that these empirical demonstrations are all with older children through to adults. It is reasonable to expect that effect sizes and the generalization of change will be considerably more impressive when they are targeted at young children during critical, or sensitive, periods for change. Thus, this study will identify more precise early intervention targets for children at risk for complex and comorbid conditions.

This study has a number of additional strengths. First, a large high-risk birth cohort will be recruited and followed for three years. Previous research using the same recruitment strategy in the same health district found that this strategy was ideal for over-sampling infants who were at high-risk for the development of mental health and developmental disorders (101). Second, by accessing reliable antenatal and birth data from participants' medical records, a range of antenatal and birth risk factors will be able to be examined and controlled. Thirdly, this study employs multi-method and multi-informant measures across time.

Although this study has several strengths, there are some limitations that must be noted. While the core features of DBD-CU, DBD+CU, excessive anxiety, ASD, and ADHD are all present and measurable by 3-years-old, there are problems with false-positives and false negatives. Unlike the other disorders, the diagnosis of ASD is relatively reliable by 3-years-old (137). There is however a proportion of children that will test positive for DBD (+/-CU), anxiety, and ADHD at 3-years-old but later desist (138). With anxiety disorders, the opposite problem may also be the case, as a substantial number of children develop a diagnosable anxiety disorder in late childhood and early adolescence. The evidence is clear that while these children may not have had a frank diagnosis at earlier ages, they will have shown long-standing problems with excessive fear, shyness, behavioral inhibition, and sensitivity, which are all traits that will be captured by our dimensional measures. False positive diagnoses and false negative diagnoses will be minimized by using multi-informant and multi-method assessment strategies that aim to produce diagnostics based on conservative and convergent data.

Another limitation is the risk of attrition. A review of longitudinal studies examining antenatal and postnatal depression found that attrition rates ranged from 6.4% - 49.9% across studies (139). Common features of non-completers across studies appeared to be mothers who were younger had lower socio-economic status, lower education, were unemployed, and those with higher depression scores (139). Although we have accounted for attrition in our power analyses, and have ensured a range of contact methods are available in order to reach our participating families over time (e.g., phone number, email address, mailing address, secondary contacts, etc.), it is possible that some families may be lost to follow up or choose to withdraw from the study. Further, it is possible that similar to other longitudinal studies, attrition may be selective. In order to assess the representativeness of the sample, we will compare key socio-demographic variables for those who drop out of the study compared to those who remain in the study at each time point.

Despite these concerns, this study has the potential to test a theoretical model of how precise variations in the REAL constructs lead to the most common childhood psychopathologies. It will help to identify the common and unique factors that underlie childhood mental health comorbidities and discrete disorders, respectively. Further, the study will identify critical variables and the developmental timings of these variables, as a road map for precise targets for early intervention for the prevalent childhood psychopathologies. Early detection of and intervention with the common childhood mental health disorders has the potential to alter adverse developmental trajectories in ways that are clinically significant for families and children, and socially and economically beneficial for communities.

## Ethics Statement

The studies involving human participants were reviewed and approved by The University of Sydney Human Research Ethics Committee (Project Number 2017/644), and the South Western Sydney Local Health District Human Research Ethics Committee (Local Project No HE17/115) have approved this study. Written informed consent was obtained from mothers for their participation and mothers also provided written informed consent for the participation of their child. Written informed consent was obtained from fathers for their participation.

## Author Contributions

FD wrote the first and successive drafts of this manuscript. All authors contributed to conception and design of the study. All authors contributed to the article and approved the submitted version.

## Funding

This publication is an outcome of the “*Watch Me Grow for REAL*” study, which is funded by the Australian Government's National Health and Medical Research Council (NHMRC; APP1127952). The funding body had no role in the study design, interpretation, writing the manuscript, or the decision to submit the paper for publication.

## Conflict of Interest

The authors declare that the research was conducted in the absence of any commercial or financial relationships that could be construed as a potential conflict of interest.

## Abbreviations

ADHD, attention deficit hyperactivity disorder; ASQ-3, Ages and Stages Questionnaire 3; ALES: Adverse Life Experiences Scale; ASD, autism spectrum disorder; CU, callous-unemotional; DASS21, Depression Anxiety Stress Scales; DBD, disruptive behavior disorders; DBD-CU, disruptive behavior disorders without accompanying elevated levels of CU traits; DBD+CU, disruptive behavior disorders with elevated levels of CU traits; DISCAP, Diagnostic Interview Schedule for Children; EDA, electro-dermal activity; JA, joint attention; M-CHAT, Modified Checklist for Autism in Toddlers; NBQ, Newborn Baseline Questionnaire; PAFAS, Parenting and Family Adjustment Scales; PAS-R, Revised Preschool Anxiety Scale; REAL, Responsiveness, Emotional Attention, and Learning; SDQ, Strengths and Difficulties Questionnaire; SWCh, Satisfaction with Childbirth; T1, Time point 1; T2, Time point 2; T3, Time point 3; T4, Time point 4.
